# Mapping of Alzheimer’s disease related data elements and the NIH Common Data Elements

**DOI:** 10.1186/s12911-024-02500-8

**Published:** 2024-04-19

**Authors:** Xubing Hao, Rashmie Abeysinghe, Fengbo Zheng, Paul E. Schulz, The Alzheimer’s Disease Neuroimaging Initiative, Licong Cui

**Affiliations:** 1 McWilliams School of Biomedical Informatics, University of Texas Health Science Center at Houston, Houston, TX USA; 2 Department of Neurology, McGovern School of Medicine, University of Texas Health Science Center at Houston, Houston, TX USA

**Keywords:** Alzheimer’s disease, Data element mapping, Semantic interoperability

## Abstract

**Background:**

Alzheimer’s Disease (AD) is a devastating disease that destroys memory and other cognitive functions. There has been an increasing research effort to prevent and treat AD. In the US, two major data sharing resources for AD research are the National Alzheimer’s Coordinating Center (NACC) and the Alzheimer’s Disease Neuroimaging Initiative (ADNI); Additionally, the National Institutes of Health (NIH) Common Data Elements (CDE) Repository has been developed to facilitate data sharing and improve the interoperability among data sets in various disease research areas.

**Method:**

To better understand how AD-related data elements in these resources are interoperable with each other, we leverage different representation models to map data elements from different resources: NACC to ADNI, NACC to NIH CDE, and ADNI to NIH CDE. We explore bag-of-words based and word embeddings based models (Word2Vec and BioWordVec) to perform the data element mappings in these resources.

**Results:**

The data dictionaries downloaded on November 23, 2021 contain 1,195 data elements in NACC, 13,918 in ADNI, and 27,213 in NIH CDE Repository. Data element preprocessing reduced the numbers of NACC and ADNI data elements for mapping to 1,099 and 7,584 respectively. Manual evaluation of the mapping results showed that the bag-of-words based approach achieved the best precision, while the BioWordVec based approach attained the best recall. In total, the three approaches mapped 175 out of 1,099 (15.92%) NACC data elements to ADNI; 107 out of 1,099 (9.74%) NACC data elements to NIH CDE; and 171 out of 7,584 (2.25%) ADNI data elements to NIH CDE.

**Conclusions:**

The bag-of-words based and word embeddings based approaches showed promise in mapping AD-related data elements between different resources. Although the mapping approaches need further improvement, our result indicates that there is a critical need to standardize CDEs across these valuable AD research resources in order to maximize the discoveries regarding AD pathophysiology, diagnosis, and treatment that can be gleaned from them.

## Background

Alzheimer’s Disease (AD) is a degenerative neurological condition that impairs memory and thinking skills, and has become a public health crisis. The cost of AD to society is substantial as AD patients require significant expenditures for health care, intensive long-term services, and support. In the US, the total health care costs for AD treatment in 2020 is estimated at $305 billion [1].

The US National Alzheimer’s Project Act (NAPA) is a National Plan for Alzheimer’s disease and related dementias (AD/ADRD) that mobilizes public and private resources with the goal of preventing and effectively treating AD and ADRD by 2025. The AD+ADRD Research Implementation Milestones detail specific steps and success criteria towards achieving the goal of NAPA, and have eight focus areas including *Enabling Infrastructure*. *Data Sharing and Reproducibility* is one of the subareas under *Enabling Infrastructure*, and further contains seven specific milestones (Milestones 3.A – 3.G). Milestone 3.A aims at providing resources to make datasets from high value, publicly funded clinical research/cohort studies widely accessible, (re)usable and interoperable.

Major strides in data sharing for AD research in the US include the National Alzheimer’s Coordinating Center [NACC] [2] and the Alzheimer’s Disease Neuroimaging Initiative [ADNI] [3], which provide valuable resources for discoveries regarding AD pathophysiology, diagnosis, and treatment. On the other hand, the US National Institutes of Health (NIH) has launched NIH Common Data Elements (CDE) Repository to provide open access to structured definitions of data elements recommended or required by NIH Institutes and Centers or other organizations, in order to facilitate the interoperability among data sets in various disease research areas including neurological conditions. However, the extent to which these three resources (NACC, ADNI, and NIH CDE) are interoperable with each other with regard to AD-related data elements is unclear.

The goal of this work is to map data elements among NACC, ADNI and NIH CDEs in order to better understand their interoperability between each other. We explore and compare bag-of-words and word embedding models to perform the data element mappings between different resources.

### National Alzheimer’s coordinating center

NACC has developed and maintains a large database of more than 156,000 participants’ information from Alzheimer’s Disease Research Centers (ADRCs) funded by the National Institute on Aging [4]. The NACC database includes the longitudinal clinical data, neuropathological data, imaging and fluid biomarker data, and genetic data. The Uniform Data Set (UDS) is the primary data set for researchers analyzing clinical and demographic data.

### Alzheimer’s disease neuroimaging initiative

ADNI is a collaborative effort supported by the NIH and private sector to develop clinical, imaging, genetic, and biochemical biomarkers for early detection and tracking of AD [3]. ADNI data have been widely used by researchers around the world, which has resulted in over 2,100 publications. The study data shared by ADNI involve subject demographics, family history, diagnosis and neuropsychological assessments, biospecimen data, genetic results, MRI and PET images, medical history, and neuropathology results.

### NIH Common Data Elements

CDEs are standardized, precisely defined questions (or variables) paired with a set of specific allowable responses (or permissible values), used across different sites or studies [5]. Use of CDEs can facilitate data sharing and standardization to improve data quality and enhance research reproducibility, as well as enable data harmonization and integration from multiple sources, including electronic health records.

NIH has encouraged the use and development of CDEs in patient registries, clinical studies, and other human subjects research to improve accuracy, consistency, and interoperability among data sets within various areas of health and disease research. The NIH CDE Repository, hosted by the National Library of Medicine, has integrated a total of more than 27,000 CDEs, including over 18,000 elements from the National Institute of Neurological Disorders and Stroke (NINDS) and over 1,500 elements from the National Cancer Institute (NCI).

### Data element mapping

Mapping data elements across different sources has been an active research area in biomedical data integration. For instance, Mougin et al. performed data element mapping from a collection of gene-related, protein-related, and disease-related sources to the National Cancer Institute (NCI) Cancer Data Standards Registry (caDSR) and the Unified Medical Language System (UMLS) [6]. Pathak et al. mapped phenotype data elements from five eMERGE (Electronic Medical Records and Genomics) Network sites to the caDSR and SNOMED CT [7]. Liu et al. mapped data elements in the Dental Information Model (DIM) to the caDSR common data elements [8]. To the best of our knowledge, this paper is the first study to map AD-related data elements.

## Methods

In this work, bag-of-words and word embeddings models are leveraged to map AD-related data elements between NACC, ADNI and NIH CDE Repository. The mapping results are manually reviewed by human experts. For valid mappings, the consistency of permissible value is further examined.

### Materials

**Fig. 1:**
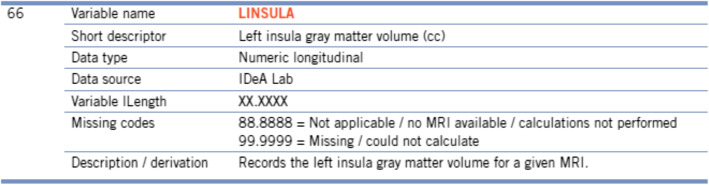
An example data element from NACC’s imaging data dictionary in PDF. The form name of this data element is “*Imaging*”. The short descriptor of this data element is “*Left insula gray matter volume (cc)*”

**Fig. 2:**
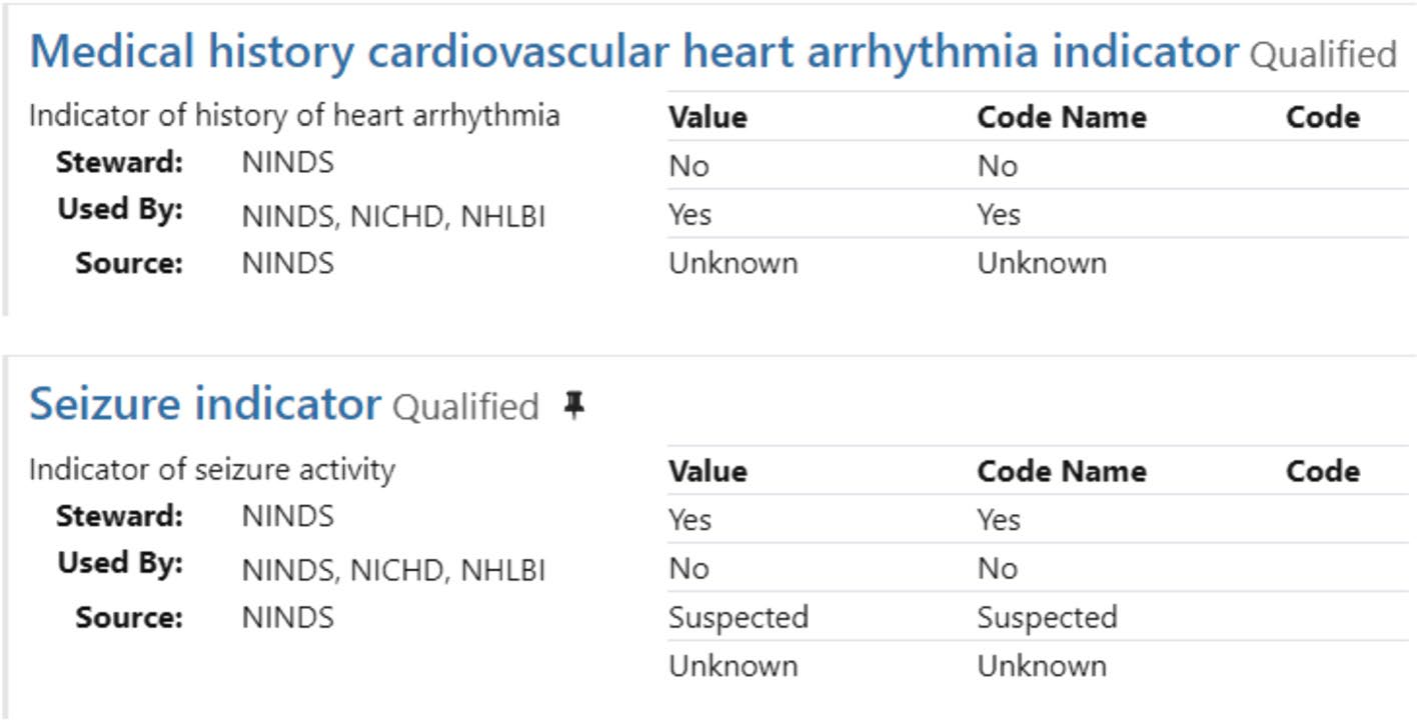
Two examples of common data elements in NIH CDE Repository

We downloaded structured data dictionaries (in CSV and PDF) from NACC, ADNI and NIH CDE Repository on November 23, 2021. The CSV data dictionaries provided by NACC contain data elements in the UDS, Neuropathology (NP) data set, and genetic data. The PDF data dictionaries provided by NACC contain data elements in the imaging and biomarker data sets. Figure 1 shows an example data element in NACC’s imaging data dictionary. We used the open-source *pdftotext* utility (part of the *Xpdf* software suite [9]) to convert PDF data dictionaries to plain text files, which were further parsed to extract attributes of data elements and store them in CSV. Figure 2 shows two examples of data elements in NIH CDE Repository.

For data element mapping, we leverage “Form” and “Short descriptor” of NACC data elements, “CRF NAME” and “TEXT” of ADNI data elements, as well as “Name” and “Question Texts” of data elements in NIH CDE Repository.

### Data element preprocessing

For NACC and ADNI, since the same data element may be collected in different study phases or visits (i.e., the same information being captured multiple times), we keep one instance and remove duplicated ones for mapping. Table 1 shows an example of such a data element in ADNI, which was captured in four phases (i.e., ADNI1, ADNIGO, ADNI2, and ADNI3). We further filter out ambiguous data elements containing “other” and “specify”, because they are always associated with another data element and likely to cause incorrect mappings. For instance, data element “*Other, specify*” in NIH CDE Repository may relate to “*Tremor type*”, “*Employment current status code*”, or “*Primary caregiver relation patient type*”, etc. We also disregarded a few ADNI data elements which only have “CRF NAME” but lack of “TEXT”.

**Table 1:** Example of duplicated data elements in ADNI

Phase	FLDNAME	TBLNAME	CRFNAME	TEXT	TYPE	LENGTH	CODE	UNITS
ADNI1	HMSTEPWS	MODHACH	Modified Hachinski	2. Stepwise Deterioration of Dementia	N	1	1=Present - 1 point; 0=Absent	
ADNIGO	HMSTEPWS	MODHACH	Modified Hachinski	2. Stepwise Deterioration of Dementia	N	1	1=Present - 1 point; 0=Absent	
ADNI2	HMSTEPWS	MODHACH	Modified Hachinski	2. Stepwise Deterioration of Dementia	N	1	1=Present - 1 point; 0=Absent	
ADNI3	HMSTEPWS	MODHACH	Modified Hachinski	2. Stepwise Deterioration of Dementia	N		0=Absent;1=Present - 1 point	

Furthermore, in NACC’s CSV data dictionaries, the forms are using short names (e.g., “a1”, “a2”, “b6”). We found the full names of the forms through NACC’s website, and converted the short names to full names for NACC’s data dictionaries. For example, form “b6” has a full name of “Behavioral Assessment - Geriatric Depression Scale”. In this paper, we use the full names of the forms when describing examples. In ADNI’s data dictionary, for some imaging-related data elements, words in phrases describing brain regions are concatenated without spaces. We pre-process such cases and add spaces between the concatenated words. For example, after pre-processing “*Cortical Thickness Average of RightTemporalPole*” we obtain “*Cortical Thickness Average of Right Temporal Pole*”.

In addition, we normalize the text describing data elements before performing the mapping. The normalization consists of the following three steps: (1) convert the text to be lowercase, and remove punctuations like [! ” $$\#$$ $ $$\%$$] as well as extra whitespaces in the text; (2) filter out stop words (such as “a”, “the”, “on”, and “is”) from the text using the open-source Natural Language Toolkit (NLTK) in Python [10]; and (3) perform lemmatization using WordNet Lemmatizer in NLTK. For instance, both the short descriptor of NACC data element “*Body bradykinesia and hypokinesia*” and the name of NIH CDE “*Body bradykinesia - hypokinesia*” are normalized to “body bradykinesia hypokinesia”.

### Bag-of-words based mapping

For bag-of-words based mapping, we model each data element as a bag-of-words. Given two data elements from different sources, we calculate the cosine similarity score of the bags-of-words of these two data elements. For instance, the cosine similarity between the NACC data element with form name of “*Behavioral Assessment - Geriatric Depression Scale*” and short descriptor of “*Are you basically satisfied with your life?*” and ADNI data element with CRF name of “*Geriatric Depression Scale*” and text of “*1. Are you basically satisfied with your life?*” is 0.9. Note that the form name and short descriptor of NACC are combined as “*Behavioral Assessment - Geriatric Depression Scale Are you basically satisfied with your life?*”, and the CRF name and text of ADNI are combined as “*Geriatric Depression Scale 1. Are you basically satisfied with your life?*” when conducting the mapping process.

We consider data elements with a cosine similarity score of above 0.6 as mapped ones for further review and evaluation. We perform the data element mappings between different sources as follows: NACC to ADNI, NACC to NIH CDE, and ADNI to NIH CDE. If a data element from source *A* has multiple mapped data elements in source *B*, we only keep the one with the highest similarity score.

### Word embeddings based mapping

For word embeddings based mapping, we explore word embeddings obtained by two widely used pre-trained models, Word2Vec [11–13] and BioWordVec [14], to generate word vectors for data elements. Word2Vec model has been trained on the Google News dataset. BioWordVec combines subword information from unlabeled biomedical text and Medical Subject Headings (MeSH). BioWordVec provides two kinds of word embeddings: “Bio-embedding-intrinsic” for calculating semantic similarity between words, terms or sentences; and “Bio-embedding-extrinsic” used as the input for downstream tasks such as relation extraction or text classification. In this work, we leverage “Bio-embedding-intrinsic” word embeddings, since our task is to compute the semantic similarity of data elements.

The Word2Vec and BioWordVec models adopted in this work are both pre-trained embedding models with default settings and parameters. For instance, in Word2Vec, *vector_size* with a default value of 100 is the dimensionality of the word vectors; *alpha* with a default value of 0.025 is the initial learning rate; *window* with a default value of 5 is the maximum distance between the current and predicted word within a sentence; *min_count* with a default value of 5 means ignoring words with total frequency lower than 5; and *sample* with a default value of 0.001 is the threshold for configuring which higher-frequency words are randomly downsampled. In BioWordVec, the vector size is 200; the learning rate is 0.05; the window size is set to be 20; *min_count* is 5; and the sampling threshold is 0.0001.

To measure the similarity of two data elements based on their embeddings, we leverage the Word Mover’s Distance (WMD) method [15], which is available in an open-source Python library called Gensim [16]. For example, the NACC data element with form name of “*Neuropathology*” and short descriptor of “*Paraffin-embedded blocks of brain regions*” and the NIH CDE with a name of “*Brain tissue paraffin-embedded indicator*” and question texts of “*Are paraffin-embedded blocks of brain tissue accessible?*” have a WMD similarity of 0.65. Similar to the bag-of-words based mapping, we consider pairs of data elements with a similarity score of above 0.6 as the mapping candidates for evaluation.

### Evaluation and value mapping

To compare the effectiveness of the bag-of-words and word embedding based models, we manually review and evaluate the mapping results between data elements in different sources. For each pair of data elements evaluated as a correct mapping, we further examine their value types (e.g., numerical, categorical, date, and text) and permissible values for further mapping. For mapped data elements with a numerical type, we check if they share the same unit; and for mapped data elements with a categorical type, we check if the permissible values are completely identical, or partially identical, or different.

## Results

The data dictionaries that we downloaded contain 1,195 NACC data elements, 13,918 ADNI data elements, and 27,213 NIH CDEs. After the data element preprocessing, the numbers of NACC and ADNI data elements were reduced to 1,099 and 7,584 respectively.

### Data element mapping

For the bag-of-words based approach (see Table 2), 156 pairs of mapped data elements from NACC to ADNI were identified, and 77 of them were evaluated as valid (or correct) mappings; 228 pairs from NACC to NIH CDE were identified and 73 of them were valid; and 382 pairs from ADNI to NIH CDE were identified and 90 of them were valid.

**Table 2:** Mapping result of the bag-of-words approach

Mapping resources	Valid mappings (Percentage)	Invalid mappings (Percentage)	Total mappings
NACC $\rightarrow$ ADNI	77 (49.36%)	79 (50.64%)	156
NACC $\rightarrow$ NIH CDE	73 (32.02%)	155 (67.98%)	228
ADNI $\rightarrow$ NIH CDE	90 (23.56%)	292 (76.44%)	382

For the Word2Vec based approach (see Table 3), 287 pairs of mapped data elements from NACC to ADNI were obtained, among which 120 were evaluated as valid mappings; 223 pairs from NACC to NIH CDE were obtained and 63 of them were valid; and 545 pairs from ADNI to NIH CDE were obtained and 80 of them were valid.

**Table 3:** Mapping result of the Word2Vec approach

Mapping resources	Valid mappings (Percentage)	Invalid mappings (Percentage)	Total mappings
NACC $\rightarrow$ ADNI	120 (41.81%)	167 (58.19%)	287
NACC $\rightarrow$ NIH CDE	63 (28.25%)	160 (71.75%)	223
ADNI $\rightarrow$ NIH CDE	80 (14.68%)	465 (85.32%)	545

For the BioWordVec based approach (see Table 4), 633 mapping pairs of data elements from NACC to ADNI were identified and 164 of them were evaluated as valid; 765 pairs from NACC to NIH CDE were identified and 89 of them were valid; and 2,448 pairs from ADNI to NIH CDE were identified and 150 of them were valid.

**Table 4:** Mapping result of the BioWordVec approach

Mapping resources	Valid mappings (Percentage)	Invalid mappings (Percentage)	Total mappings
NACC $\rightarrow$ ADNI	164 (25.91%)	469 (74.09%)	633
NACC $\rightarrow$ NIH CDE	89 (11.63%)	676 (88.37%)	765
ADNI $\rightarrow$ NIH CDE	150 (6.13%)	2,298 (93.87%)	2,448

**Fig. 3:**
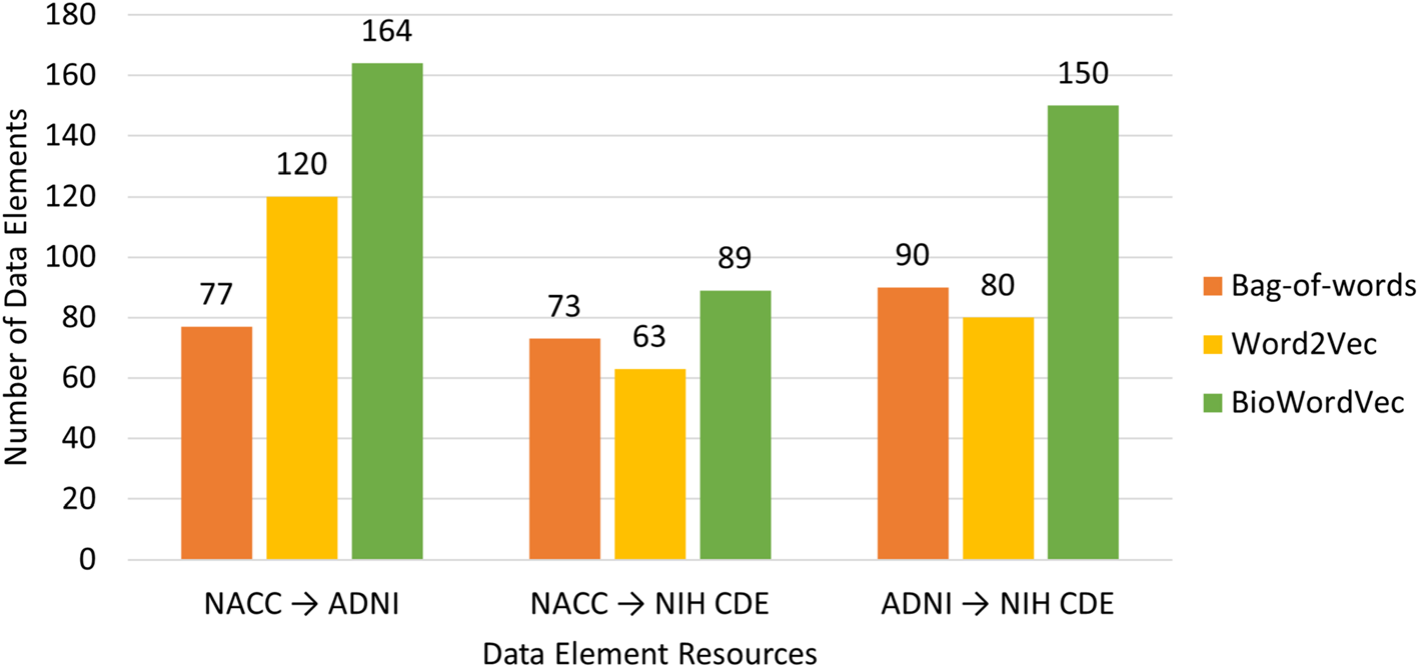
Comparison of numbers of valid mappings identified among three resources by different approaches

Figure 3 displays a bar graph comparing the number of valid mappings obtained by each approach among three resources. Overall, the BioWordVec approach identified more numbers of valid mappings than the bag-of-words approach and Word2Vec approach.

Table 5 lists nine examples of valid mappings identified by different approaches. For instance, the bag-of-words approach identified a valid mapping from the NACC data element with form name of “*Unified Parkinson’s Disease Rating Scale (UPDRS)*” and short descriptor of “*Finger taps - right hand*” to the NIH CDE with name of “*Movement Disorder Society - Unified Parkinson’s Disease Rating Scale (MDS UPDRS) - finger tap right hand score*” and question texts of “*FINGER TAPPING*”.

**Table 5:** Examples of valid mappings identified by three approaches

Approach	Mapping resources	Data element 1	Data element 2	Similarity
Bag-of-words	NACC $\rightarrow$ ADNI	**Form:** Neuropathology	**CRF NAME:** NACC Neuropathology Data Form	0.86
		**Short descriptor:** Medial temporal lobe sclerosis present (including hippocampal sclerosis)	**TEXT:** Is medial temporal lobe sclerosis (including hippocampal sclerosis) present?	
Bag-of-words	NACC $\rightarrow$ NIH CDE	**Form:** Unified Parkinson’s Disease Rating Scale (UPDRS)	**Name:** Movement Disorder Society - Unified Parkinson’s Disease Rating Scale (MDS UPDRS) - finger tap right hand score	0.81
		**Short descriptor:** Finger taps - right hand	**Question Texts:** FINGER TAPPING	
Bag-of-words	ADNI $\rightarrow$ NIH CDE	**CRF NAME:** Geriatric Depression Scale	**Name:** Geriatric Depression Scale (GDS) - feel helpless indicator	0.88
		**TEXT:** 8. Do you often feel helpless?	**Question Texts:** Do you often feel helpless?	
Word2Vec	NACC $\rightarrow$ ADNI	**Form:** Imaging	**CRF NAME:** Longitudinal FreeSurfer	0.66
		**Short descriptor:** Left fusiform mean cortical thickness (mm)	**TEXT:** Cortical Thickness Average of LeftFusiform	
Word2Vec	NACC $\rightarrow$ NIH CDE	**Form:** Behavioral Assessment - Geriatric Depression Scale	**Name:** Geriatric Depression Scale (GDS) - people better off indicator	0.78
		**Short descriptor:** Do you think that most people are better off than you are?	**Question Texts:** Do you think that most people are better off than you are?	
Word2Vec	ADNI $\rightarrow$ NIH CDE	**CRF NAME:** ECG	**Name:** ECG PR interval	0.85
		**TEXT:** PR Interval	**Question Texts:** PR interval	
BioWordVec	NACC $\rightarrow$ ADNI	**Form:** Functional Assessment NACC Functional Assessment Scale	**CRF NAME:** Functional Assessment Questionnaire	0.64
		**Short descriptor:** In the past four weeks, did the subject have any difficulty or need help with: Remembering appointments, family occasions, holidays, medications	**TEXT:** 9. Remembering appointments, family occasions, holidays, medications.	
BioWordVec	NACC $\rightarrow$ NIH CDE	**Form:** Subject Health History	**Name:** Surgical history pacemaker indicator	0.63
		**Short descriptor:** Pacemaker	**Question Texts:** Pacemaker	
BioWordVec	ADNI $\rightarrow$ NIH CDE	**CRF NAME:** Mini Mental State Exam (MMSE)	**Name:** Mini-Mental State Examination (MMSE) - total score	0.82
		**TEXT:** MMSE TOTAL SCORE	**Question Texts:** MMSE total score	

**Fig. 4:**
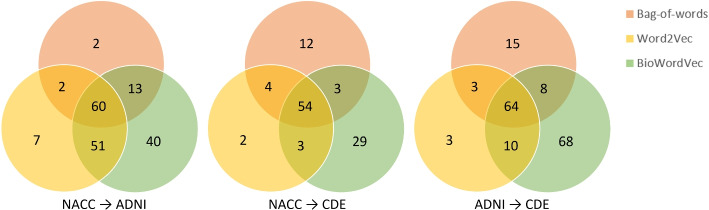
Venn diagrams of aggregated mappings among three resources. A total of 175 mappings were identified from NACC to ADNI, 107 from NACC to NIH CDE, and 171 from ADNI to NIH CDE

We further calculated the precision and recall of each approach applied to different mapping resources. Since different approaches may identify the same mapping, we aggregated the mapping results obtained by three approaches and removed duplicated mappings. From NACC to ADNI, there were a total of 720 mappings identified by three approaches, among which 175 were evaluated as valid. From NACC to NIH CDE, there were a total of 867 mappings identified by three approaches, among which 107 were evaluated as valid. From ADNI to NIH CDE, a total of 2,772 mappings were identified by three approaches, and 171 of them were evaluated as valid. Figure 4 displays the Venn diagram of the aggregated valid mappings identified by three approaches from NACC to ADNI, NACC to NIH CDE, and ADNI to NIH CDE, respectively.

**Fig. 5:**
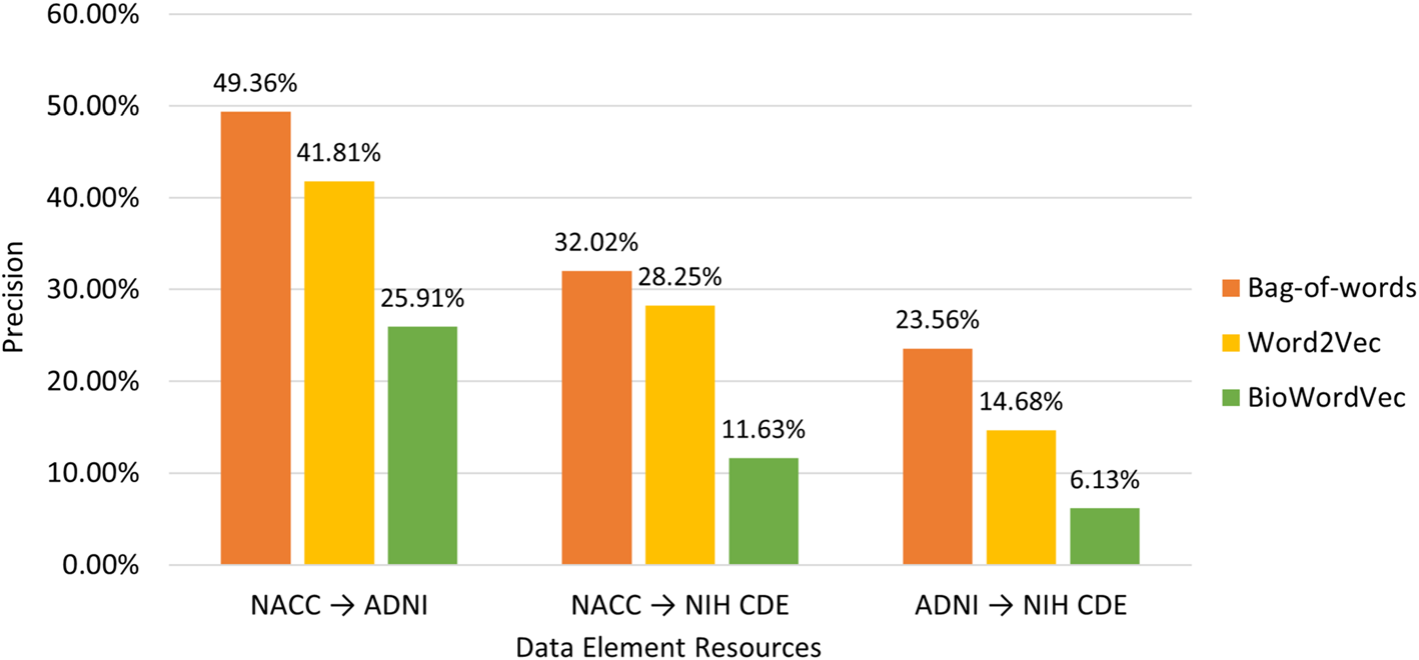
Precision of different approaches

**Fig. 6:**
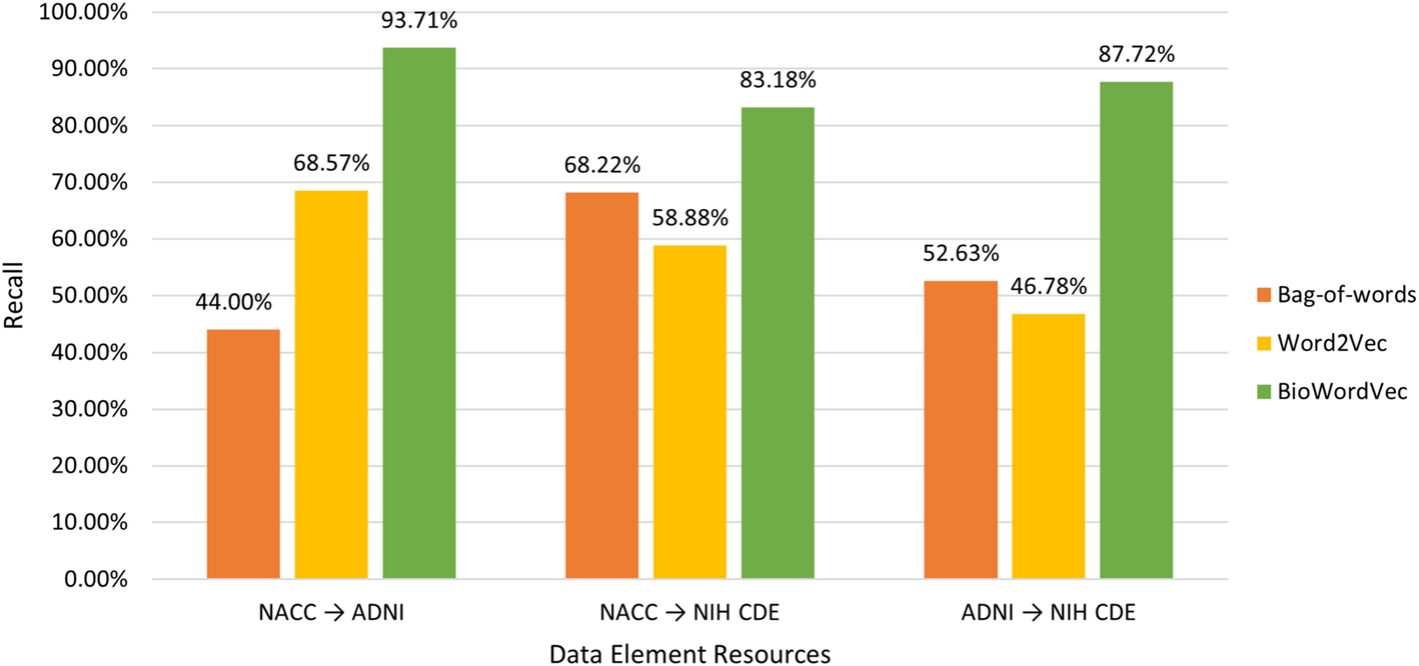
Recall of different approaches

Based on the aggregated valid mappings, we compute the precision and recall of different approaches with regard to disparate mappings resources (see Figs. 5 and 6). It can be seen that the bag-of-words approach achieved the best precision, while the BioWordVec approach attained the best recall.

### Permissible value mapping

For the mappings evaluated to be valid, we examined whether the two data elements involved in each mapping share the same value type (see Table 6). From NACC to ADNI, 173 out of 175 pairs of mapped data elements have the identical value type. From NACC to NIH CDE, 105 out of 107 pairs of mapped data elements share the same value type. From ADNI to NIH CDE, 164 out of 171 pairs of mapped data elements have the identical value type. For example, both the NACC data element with form name of “*Unified Parkinson’s Disease Rating Scale (UPDRS)*” short descriptor of “*Finger taps - right hand*” and the NIH CDE with name of “*Movement Disorder Society - Unified Parkinson’s Disease Rating Scale (MDS UPDRS) - finger tap right hand score*” and question texts as “*FINGER TAPPING*” have a numerical value type. An example of disparate value type is the mapping between the NACC data element with form name of “*Subject Health History*” and short descriptor of “*Average number of packs smoked per day*” and the ADNI data element with CRF name of “*Medical History*” and text of “*16a. During periods of smoking, the average number of packs/day*”, where the former data element has a categorical value type while the latter one has a numerical value type.

**Table 6:** Result of value type mapping

Mapping resources	Identical (Percentage)	Disparate (Percentage)	Total
NACC $\rightarrow$ ADNI	173 (98.86%)	2 (1.14%)	175
NACC $\rightarrow$ NIH CDE	105 (98.13%)	2 (1.87%)	107
ADNI $\rightarrow$ NIH CDE	164 (95.91%)	7 (4.09%)	171

For mapped data elements sharing the same value type (numerical or categorical), we further checked the consistency of their permissible values. More specifically, we compared units for numerical data elements if applicable, and value lists for categorical data elements.

For the mapped data elements of numerical type, we classified their unit consistency checking results into three categories: identical, disparate, and not available (see Table 7). For example, the NACC data element with form name of “*Imaging*” and short descriptor of “*Segmented right hippocampus volume (cc)*” and its mapped ADNI data element with CRF name of “*UCSF SNT Hippocampal Volumes*” and text of “*Right Hippocampus Volume*” have disparate measurement units: the former is “*cc*”, while the latter is “*mm3*”. If at least one of the two data elements does not provide unit information, we categorized their unit consistency result as not available.

**Table 7:** Result of unit consistency checking for mapped numerical data elements

Mapping resources	Identical (Percentage)	Disparate (Percentage)	Not available (Percentage)	Total
NACC $\rightarrow$ ADNI	63 (67.02%)	8 (8.51%)	23 (24.47%)	94
NACC $\rightarrow$ NIH CDE	2 (16.67%)	4 (33.33%)	6 (50.00%)	12
ADNI $\rightarrow$ NIH CDE	8 (44.44%)	2 (11.11%)	8 (44.44%)	18

For the mapped data elements of categorical type, we classified their value list consistency results into three categories: identical, partially identical, and disparate (see Table 8). For instance, the ADNI data element with CRF name of “*Geriatric Depression Scale*” and text of “*8. Do you often feel helpless?*” and its mapped NIH CDE with name of “*Geriatric Depression Scale (GDS) - feel helpless indicator*” and question texts of “*Do you often feel helpless?*” in Table 5 have partially identical value list. Specifically, permissible values of ADNI’s data element is a proper subset of permissible values of NIH CDE. The former has two permissible values: “*1=Yes*” and “*0=No*”; and the latter has three permissible values: “*Yes*”, “*No*”, and “*Unknown*”.

**Table 8:** Result of consistency checking of permissible values for mapped categorical data elements

Mapping resources	Identical (Percentage)	Partially identical (Percentage)	Disparate (Percentage)	Total
NACC $\rightarrow$ ADNI	14 (17.72%)	64 (81.01%)	1 (1.27%)	79
NACC $\rightarrow$ NIH CDE	5 (5.38%)	73 (78.49%)	15 (16.13%)	93
ADNI $\rightarrow$ NIH CDE	21 (17.95%)	40 (34.19%)	56 (47.86%)	117

## Discussion

In this work, we have explored bag-of-words, Word2Vec, and BioWordVec based approaches to map data elements in NACC, ADNI, and NIH CDE. In total, the three approaches mapped 175 out of 1,099 (15.92%) NACC data elements to ADNI; 107 out of 1,099 (9.74%) NACC data elements to NIH CDE; and 171 out of 7,584 (2.25%) ADNI data elements to NIH CDE. This indicates that there is a critical need to develop standardized CDEs for AD research to facilitate data sharing and interoperability of various AD-related data sets. Given the wide usage of ADNI and NACC, they may serve as valuable references for creating such standardized AD-related CDEs.

### Comparison of three approaches

Bag-of-words approach represents text as vector by a bag-of-words. The similarity between two texts is calculated by cosine distance between the vectors. When conducting our mapping task, bag-of-words approach has an advantage of being simple and not requiring very complicated processing. In our study, leveraging bag-of-words approach to map data elements costed much less time than the Word2Vec approach and BioWordVec approach. However, bag-of-words approach lacks representation between similar words and may not perform well when text has repeated words. For example, bag-of-words approach failed to identify a correct mapping of “*Subject Demographics - Marital status*” in NACC and “*Participant Demographics - 4. Participant Marital Status*” in ADNI while Word2Vec approach successfully identified it. Here, “Participant” appears twice in ADNI’s data element and brings down the similarity score of bag-of-words approach.

For Word2Vec, the word vector is a low-dimensional real number vector obtained by training, which solves bag-of-words approach’s problem of semantic deficiency due to the independence of words. Since Word2Vec takes the context into consideration, it can identify certain correct mappings that bag-of-words approach cannot. However, one disadvantage of Word2Vec is that since there is a one-to-one relationship between words and vectors, there is a problem for polysemy.

As for BioWordVec, it has shown effectiveness and utility in multiple NLP tasks in the biomedical domain. Word embeddings trained on the PubMed and PMC corpora significantly outperform those trained on Google News. It improves the quality of biomedical word representations and better capturing their semantics. For example, bag-of-words approach and Word2Vec approach failed to identify a correct mapping of “*Imaging - Left pars triangularis mean cortical thickness (mm)*” in NACC and “*Longitudinal Free Surfer - Cortical Thickness Average of Left Pars Triangularis*” in ADNI while BioWordVec successfully identified it. After further examination, we found that Word2Vec’s Google News word vectors set does not contain the vector of “*Triangularis*”, but BioWordVec’s bio word vectors set contains it. Furthermore, the similarity of “*mean*” and “*average*” in BioWordVec is 0.8524 while their similarity in Word2Vec is 0.1956. Comparing to the other two methods, BioWordVec identified the greatest number of correct mappings in this work.

### Limitations and future work

With regard to the performance of different approaches, since there was no gold standard available for the mapping results, the reported performance metrics (precision and recall) were based on the manual review and evaluation of the candidate mappings identified by the three approaches. It is possible that certain valid mappings were not obtained by any of the three approaches (i.e., missed by our approaches), indicating that the actual recall may be lower than the reported recall. Furthermore, it can be seen from Fig. 5 that the three approaches showed limited precision (ranging from 6.13% to 49.36%). These indicate that additional work is still needed to improve both precision and recall of the mapping approaches.

#### Analysis of false positive cases

For potential improvement of precision, we examined some of the false positive cases (i.e., invalid mappings) and analyzed potential reasons. One reason is that the mapping approaches were not capable of distinguishing the nuances between the data elements. For example, NACC data element with form name of “*Co-participant Demographics*” and short descriptor of “*Co-participant’s month of birth*” was mapped to ADNI data element with CRF name of “*Participant Demographics*” and text of “*2a. Participant Month of Birth*” with similarity score of 0.74, but they are targeting different subjects (*co-participant* versus *participant*). Another example is that NACC data element with form name of “*Neuropsychological Battery Scores*” and short descriptor of “*Rey Auditory Verbal Learning (Immediate) Trial 3 Total recall*” was mapped to ADNI data element with CRF name of “*Neuropsychological Battery*” and text of “*Rey Auditory Verbal Learning Test Trial 1 Total*” with similarity score of 0.73. However, NACC refers to Trial 3, while ADNI refers to Trial 1. A potential way to avoid such false positives is to assign importance weights for the different words between the two data elements and assess whether their difference is significant or not.

#### Analysis of false negative cases

For potential improvement of recall, we manually examined some data elements in NACC and ADNI and identified a few causes for false negative cases (i.e., missed mappings by three approaches). One is that our approaches did not leverage acronyms. For example, NACC uses “*APOE genotype*” while ADNI uses “*Apolipoprotein-E*”; thus this mapping was missed by our approaches. In future work, we plan to leverage the Unified Medical Language System (UMLS) to perform synonym substitution and explore to what extent this may help identify additional matching data elements between different resources.

Another scenario is that ADNI leverages some of the NACC’s neuropathology data elements, but it sometimes provides a longer description of the original NACC’s data elements. For example, ADNI’s data element with CRF name of “*NACC Neuropathology Data Form*” and text of “*Primary pathological diagnosis judged to be responsible for subject’s cognitive status - Prion-associated disease*” reuses NACC’s data element with form name of “*Neuropathology*” and short descriptor of “* Prion - associated disease - primary*”, but it has a much longer text description. Our approaches failed to identify such cases.

Additionally, some missed mappings were due to different design or organization of forms in NACC and ADNI. For example, consider NACC’s data element with form name of “*Neuropsychological Battery Scores*” and short descriptor of “*MoCA: Orientation - Place*” and ADNI’s data element with CRF name of “*MoCA*” and text of “*Place*”. While ADNI has a dedicated form for MoCA, NACC’s Neuropsychological Battery Scores form covers a wider range of tests including MoCA. Since our approaches combine the form name with description, “*Neuropsychological Battery Scores MoCA: Orientation - Place*” for NACC is much longer than “*MoCA Place*” for ADNI, leading to low similarity score. Assigning importance weights as mentioned above may help improve the precision.

Additional work is still needed to find out the remaining scenarios of valid mappings between different sources that have been missed by three approaches. Since manual review is a time-consuming and labor-intensive process, a more feasible solution is to randomly select a subset of data elements from one source and invite domain experts to manually map them to the other source. Such subsets would serve as partial gold standards to uncover valid mappings missed by our approaches and provide insight into enhancing the approaches.

## Conclusions

In this paper, we have explored three models (bag-of-words, Word2Vec, and BioWordVec) for representing and mapping data elements among NACC, ADNI, and NIH CDE Repository, in order to understand how AD-related data elements in these resources are interoperable with each other. Our results showed that the bag-of-words based approach attained the best precision, while the BioWordVec based approach achieved the best recall. Although the mapping approaches need further improvement, our result indicates a vital need to create standardized AD-related CDEs leveraging rich data elements in NACC and ADNI to enhance the interoperability of various datasets for AD research.

## Data Availability

The results for mapped data elements as well as their inconsistent value mappings are available at https://github.com/XubingHao/BMC2021_DE.

## Abbreviations

NACC: National Alzheimer’s Coordinating Center
ADNI: Alzheimer’s Disease Neuroimaging Initiative
AD: Alzheimer’s Disease
CDE: Common Data Elements
UDS: The Uniform Data Set
NP: Neuropathology
MeSH: Medical Subject Headings
WMD: Word Mover’s Distance
GDS: Geriatric Depression Scale
UMLS: Unified Medical Language System
ADO: Alzheimer’s disease ontology
